# Supplementary material for: In vivo validation of late‐onset Alzheimer's disease genetic risk factors
Source: Alzheimers Dement. 2024 Apr 30;20(7):4970–84. doi: 10.1002/alz.13840 (PMC11247676; doi:10.1002/alz.13840)
Supplement: Supplementary file 2 — Supporting information [file ALZ-20-4970-s006.pdf]

Supplemental Figure 1A

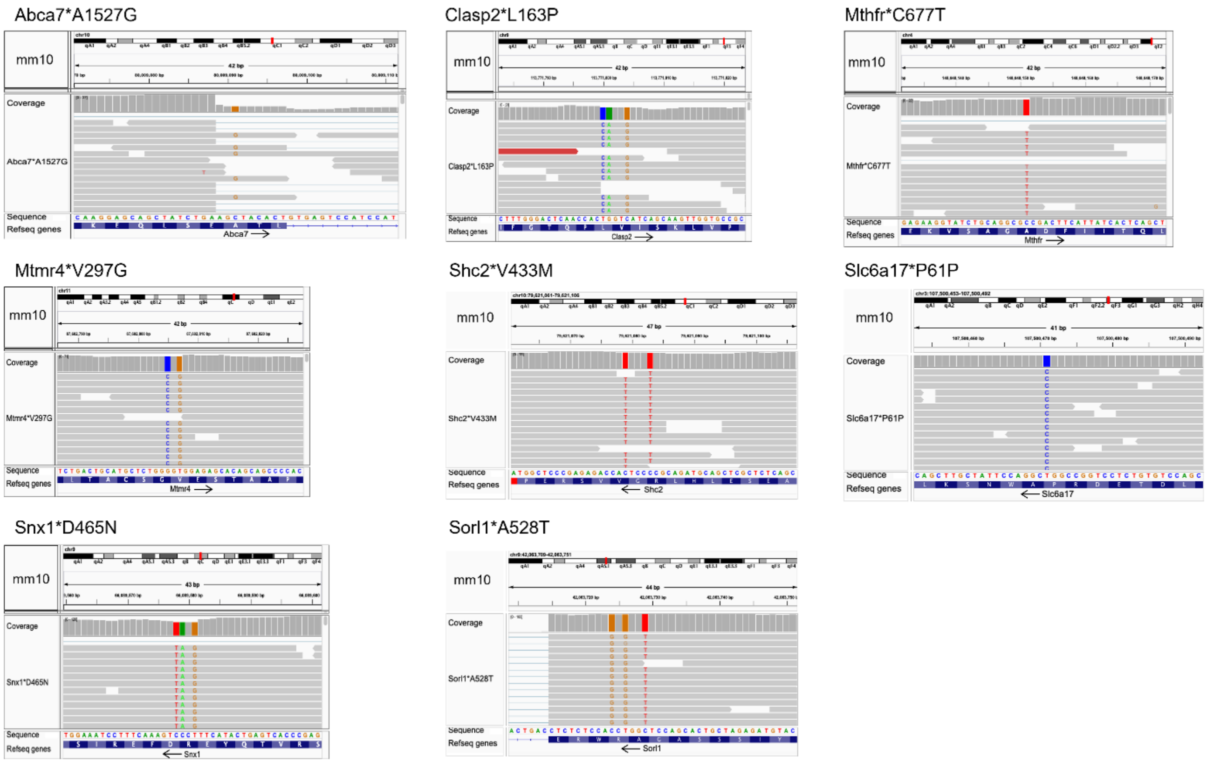

Supplemental Figure 1B

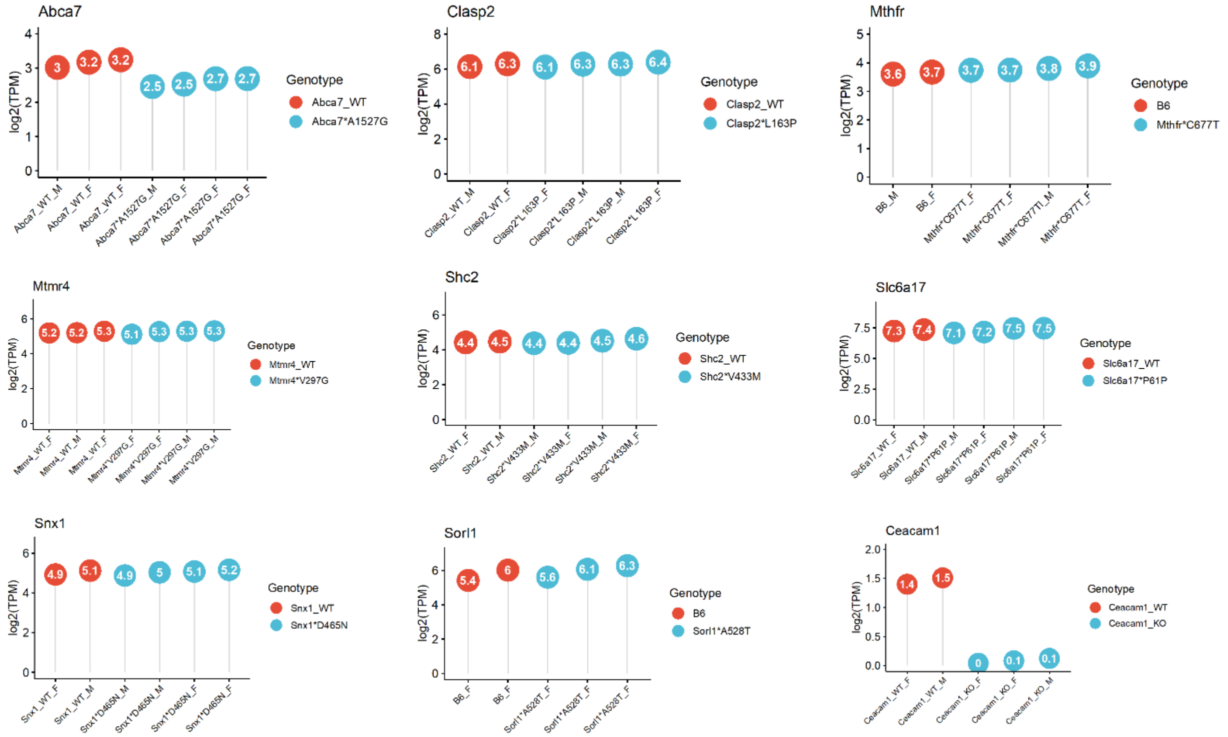

SUPPLEMENTAL FIGURE 1: Validation of novel mouse models. RNA-seq was performed on brain tissue at 4 months of age for each model. **(A)** Sequence analysis identified appropriate engineered variants; in some cases, silent mutations were introduced for CRISPR or genotyping purposes. **(B)** Transcript counts were used to demonstrate normal expression levels for SNP models, and lack of expression in the Ceacam1 knockout model.
